# Acrophyseal growth arrest in a long-term survivor of acute lymphoblastic leukemia

**DOI:** 10.1007/s00256-020-03513-w

**Published:** 2020-06-20

**Authors:** Jacky de Rooy, Stan Buckens, Paul M. Brons, Ingrid van der Geest, Filip Vanhoenacker

**Affiliations:** 1 Department of Radiology and Nuclear Medicine, Radboud University Medical Centre, Nijmegen, Netherlands; 2 Department of Pediatric Oncology, Amalia Childrens Hospital of the Radboud University Medical Centre, Nijmegen, Netherlands; 3 Department of Orthopedic Oncologic Surgery, Radboud University Medical Centre, Nijmegen, the Netherlands; 4 Department of Radiology, General Hospital Sint-Maarten, Mechelen, Universities of Antwerp and Ghent, Antwerp, Belgium

**Keywords:** Acrophysis, Growth arrest lines, Endochondral ossification, Secondary ossification center

## Abstract

Growth arrest at the secondary growth plate, also known as the acrophysis, is a rare phenomenon with only very few known published case reports. We report on a case of formation of ghost secondary ossification centers at the acrophyses of the knee joint in a 14-year-old female, who survived early childhood acute lymphoblastic leukemia. The patient suffered from severe side effects from both disease and subsequent treatment strategies with a 10-month immobilization period as a consequence at the age of 3 years. The ghost secondary ossification centers were encountered on radiographs and MRI 10 years later, when she presented for evaluation of chronic pain in her left knee related to sports activities, due to a meniscal cyst. Awareness of this phenomenon is nevertheless important, because it seems that endochondral bone growth recovery at the acrophyses might be different from recovery in physes, because we found no concomitant sequelae of growth arrest in the metaphyses.

## Introduction

Over the years, the curability of acute lymphoblastic leukemia (ALL) in children has improved to ~ 90% due to intensive therapy strategies [1]. These treatment strategies, consisting of multi-agent osteotoxic chemotherapy including also high doses of glucocorticosteroids, as well as low vitamin D levels, poor nutrition and low muscle mass, and the ALL itself, however, may all contribute to secondary, multifactorial impairment or even arrest of bone growth and significant bone morbidity [2].

Both acute and chronic skeletal abnormalities such as osteoporosis, insufficiency fractures, and osteonecrosis are among the most prevalent adverse sequelae, occurring during or shortly after finishing ALL treatment [3, 4].

Normally, altered temporal impairment of bone growth, whether caused by general illness, treatment, or local trauma, becomes evident as Harris growth arrest lines on radiographs, paralleling the epiphyseal growth plate in the proximal and distal metaphyses and diaphyses of rapid growing long bones [5]. These lines reflect a slowdown in normal endochondral ossification resulting in dense, more transversely oriented bony trabeculae [6, 7].

Endochondral ossification takes place not only at the zone of provisional calcification in the primary growth plate also known as physis, but also in the same zone surrounding the secondary ossification center (SOC) in the secondary growth plate, also known as the acrophysis. The acrophysis encompasses not only the epiphysis, but also the apophyses and carpal and tarsal centers as well as sesamoids such as the patella [8]. Therefore, growth arrest lines may appear not only in metaphyses, but also in the SOC, paralleling the hemispherical form of the SOC, thus representing a “ghost” of the SOC as it was at the time of the insult to normal endochondral bone growth. To the best of our knowledge, we report the first case of persistent ghosts of the acrophysis of the distal femur, proximal tibia, and patella in a long-term survivor of acute lymphoblastic leukemia (ALL) probably caused by underlying multifactorial causes of bone morbidity.

### Case

**Fig. 1:**
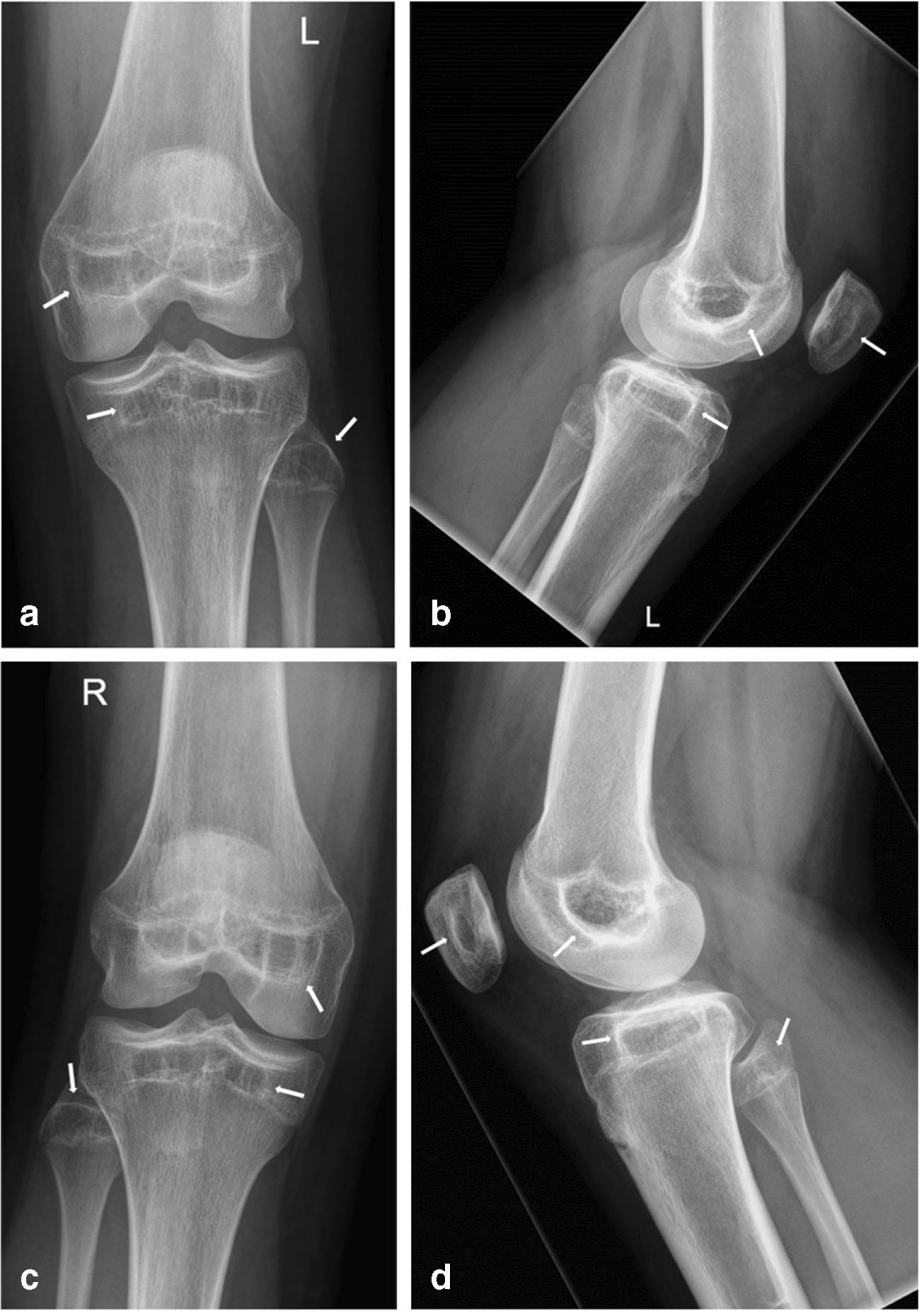
**a**–**d** PA weight-bearing and lateral conventional radiographs of both knees. Arrows indicate epiphyseal/acrophyseal growth arrest lines, outlining the former SOC

**Fig. 2:**
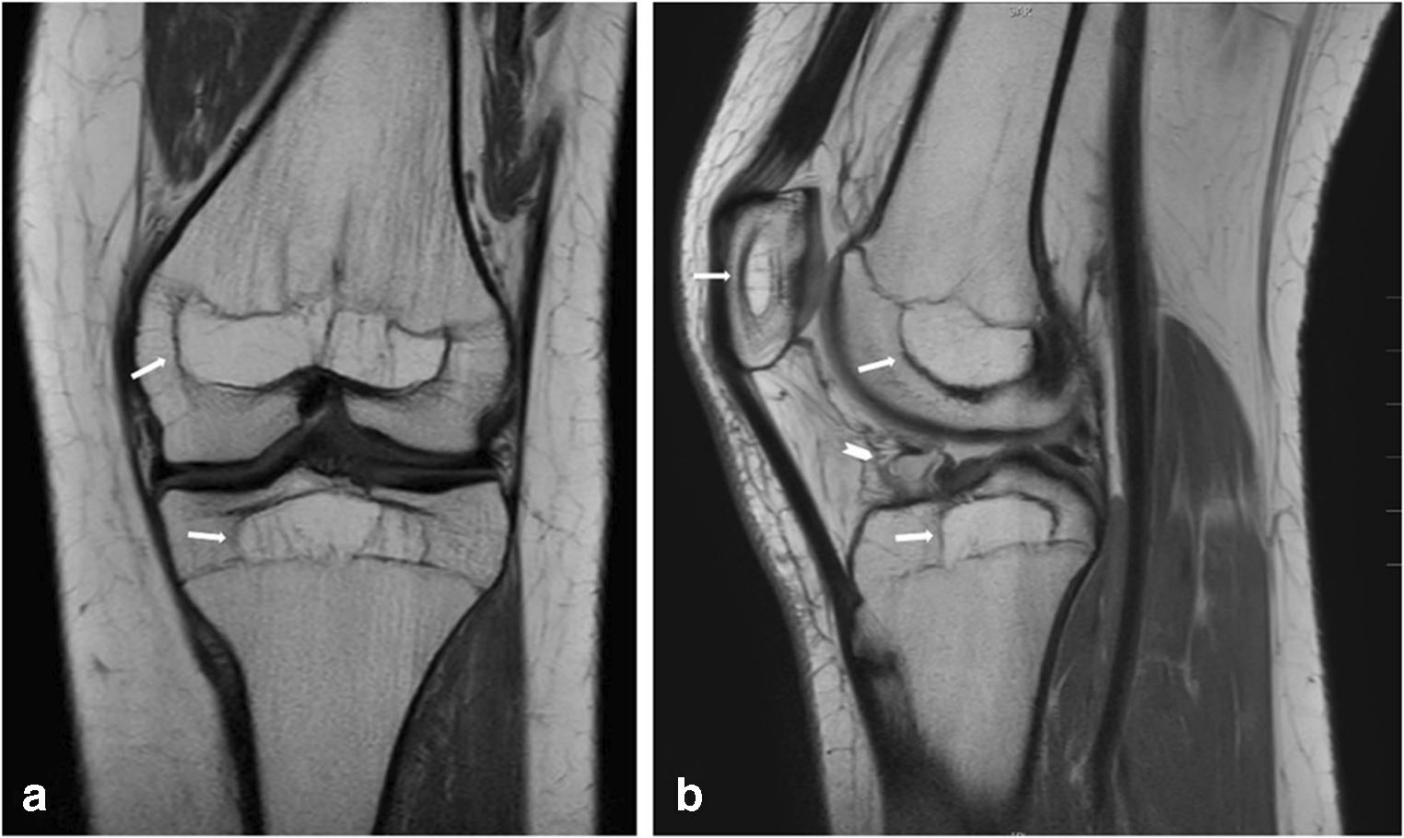
**a**–**b** MRI of the left knee. **a** Coronal T1-weighted and **b** sagittal PD-weighted images. The former SOCs are clearly delineated by low signal intensity lines in the epiphyses of the distal femur, proximal tibia, and patella; all SOCs display higher internal signal intensity than surrounding bone marrow with sparse trabeculation. Notice the absence of low signal intensity growth recovery/arrest lines in the metaphyses

A 14-year-old Caucasian female consulted our department of orthopedic surgery for a second opinion on chronic pain at the lateral side of her left knee existing for 6 months, preventing her from further participation in sports activities at school. Conventional radiographs (CR) of both knees showed sclerotic delineated radiolucencies with sparse intralesional trabeculae in the epiphyses of both knees and the patellae, following the contour of the former shape of the SOC of a, at a rough estimate, 3- to 4-year-old child (Fig. 1). A subsequent MRI of her left knee was performed to rule out intra-articular pathology. The presence of a meniscal cyst at the anterior horn of the lateral meniscus could explain the pain in the knee. In addition, a line of low signal delineating the former SOC of the epiphyses and patella was seen on T1-weighted and proton density, non-fat saturated images, abutting the still not completely closed primary growth plate of all bones. The former SOC had clearly higher internal fat signal intensity than the surrounding epiphysis on T1-weighted and PD images and displayed only sparse internal trabeculation (Fig. 2). Neither CR nor MRI showed any signs of persistent metaphyseal growth arrest/recovery lines or other additional bone pathology.

The patient had an extensive past medical history. At the age of 3, she suffered from pre-B-ALL, non-high-risk, treated with a standard multi-agent Dutch Child Oncology Group-ALL10 protocol, including vincristine, L-asparaginase, and high doses of dexamethasone with additional intrathecal triple therapy resulting in complete remission at day 40. Unfortunately, she developed a syndrome of inappropriate antidiuretic hormone secretion (SIADH) as a side effect of vincristine and treatment was further complicated by pneumonias and therapy-resistant candidiasis of the liver resulting in a long stay at the intensive care unit as a result. Due to these severe side effects, it was decided to turn away from the standard protocol after day 40 and switch to maintenance therapy with additional mercaptopurine and methotrexate, which was stopped 2 years after diagnosis. She was bedridden during at least 5 months after the onset of ALL, and her medical records did not show any increase in her length and weight during an even longer period of 10 months.

Neither brain radiation nor stem cell therapy was part of the therapy regimen. A chest radiograph showed collapse of several thoracic vertebrae at diagnosis and despite calcium and vitamin D supplementation, she suffered from an insufficiency fracture of her left femur as a result of stumbling, 8 months after diagnosis of ALL. A dual-energy X-ray absorptiometry (DXA) scan was then performed and showed a bone mineral density (BMD) of 0.271 g/cm^2^ at the level of the L1 to L4 vertebrae, with a *Z*-score of − 4.4, consistent with a very severe loss of BMD. The femur neck could not be measured at that time. Unfortunately, at 14 and 20 months after diagnosis she represented with insufficiency fractures of her right femur and both lower legs respectively, all as a result of minor traumatic events. Soon after the second event, intravenous pamidronate (bisphosphonate) therapy at a dose of 3 mg/kg was started at an interval of once per 3 months. A new DXA, performed after almost 2 years of therapy, showed a clear increase of the lumbar BMD to 0.533 g/cm^2^ with a *Z*-score of 0.5, consistent with a BMD increase of 96.5% and the pamidronate was stopped. Despite all complications, the ALL responded well to the therapy strategy and fortunately she has been disease free ever since. At the age of 10, she underwent a thorough medical control on long-term effects of childhood cancer, with complete normal outcomes as a result, especially concerning length, weight, and calcium and vitamin D levels. Further history was unremarkable until the pain in her left knee prevented her from attending sports activities at the age of 13 and urged her to visit an orthopedic surgeon.

Formal oral and written permission to publish this case was obtained from the patient and her parents.

## Discussion

From early embryonic development until closure of the growth plates, membranous and endochondral bone growths are the two main types of ossification. In membranous bone growth, bone directly arises from a membrane, consisting of sheaths of mesenchymal cells. This type of bone growth generally takes place in flat bones, but also occurs from periosteum on the surface of the diaphysis of long bones and from the peripheral fibrochondrous ring encircling the primary growth plate. In endochondral bone growth, cartilage is gradually replaced by bone at places where cartilaginous growth plates have been formed. [9]

In children, a primary cartilaginous flattened growth plate and a secondary, more (hemi)spherical cartilaginous growth plate are responsible for longitudinal bone growth by endochondral ossification. Both plates are located at the end of all long bones. [10]

**Fig. 3:**
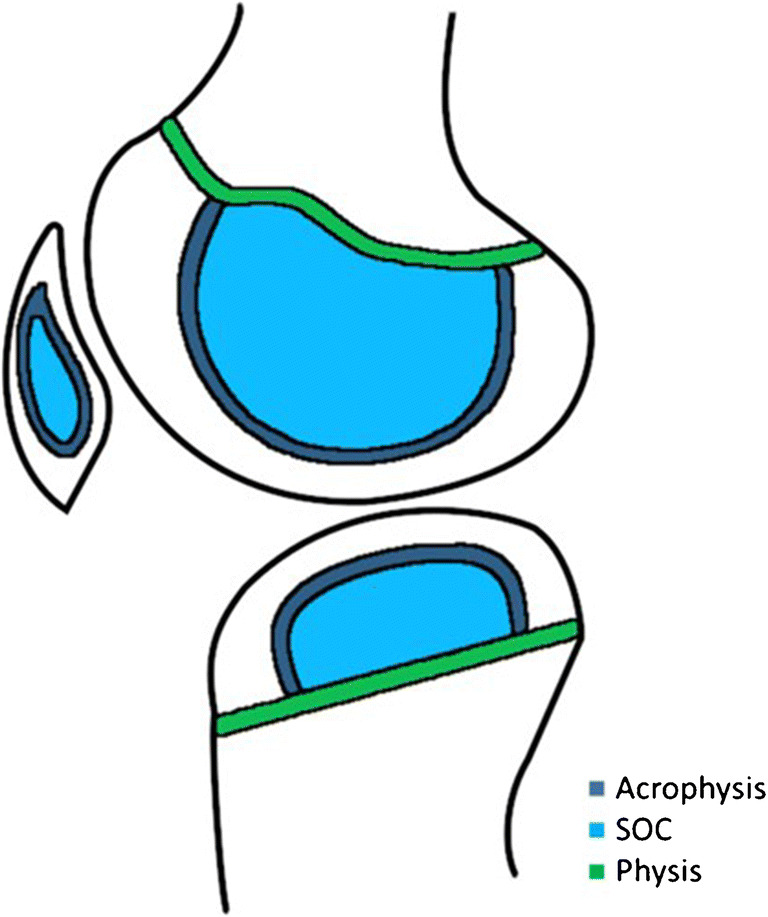
Schematic drawing of the knee representing the physes, acrophyses, and secondary growth centers (SOC) in our patient at the age of 3 years old

Endochondral bone growth responsible for an increase in length almost exclusively occurs at the primary growth plate, also called physis. The secondary growth plate, also known as acrophysis, causes multidirectional growth of the SOC within (Fig. 3). The term acrophysis was coined by Oestrich to describe all growth plates in the skeleton other than physes because of their location at the utmost extremity of bones. The term acrophysis is therefore reserved for growth plates that surround epiphyses and their apophyseal equivalents, tarsals, carpals, and sesamoids [8].

Both the primary and the secondary growth plates display a roughly identical zonal arrangement of chondrocytes, which, in order of chondral morphology and maturation, consists of a resting zone or germinal layer, followed by a columnar zone of proliferating chondrocytes and subsequently a hypertrophic zone, consisting of enlarged chondrocytes, which also secrete a mineralized extracellular matrix. Finally, these chondrocytes die and the remaining matrix is replaced by differentiated osteoblasts building up trabecular bone in the provisional zone of calcification zone [11].

As a result of a local traumatic event or systemic conditions such as severe infection, malnutrition, malignant diseases, chemotherapy, or long-term immobilization, bone growth may slow down or even stop in children [7]. The growth plate significantly narrows and the newly formed bone cannot form a normal architecture of longitudinally oriented trabeculae. Instead, trabeculae remain accumulated, pack together, and tend to form a thickened transversely oriented layer in the metaphysis, reflecting a slowdown of endochondral ossification inactivity of the primary growth plate [6]. These metaphyseal layers may become apparent on conventional radiographs as thin sclerotic growth arrest lines, also known as Harris or Park lines, once longitudinal growth is resumed [5, 6]. The longer the period of growth disturbance is, the thicker the layer. Analogously, growth arrest may occur at the acrophysis following the contour of the SOC, resulting in ghost ossification centers once the growth resumes [8].

In case of our patient, the ALL and osteotoxic treatment strategy, complicated by severe infections and a prolonged period of 10 months of inactivity, caused significant bone morbidity including severe loss of BMD accompanied with multiple insufficiency fractures and evidence of total lack of height gain at the age of 3 years [2, 12]. Our patient did not receive cranial radiation or stem cell therapy, so potential long-term effects on bone of these treatments could be safely ruled out [2, 13]. The thick sclerotic lines as seen on CR in the epiphyses and patellae of both knees 11 years after the insult might well be consistent with persistent growth arrest lines. Even more clearly, corresponding low signal intensity lines on T1-WI and PD MRI were seen outlining the contours of the former SOCs. This phenomenon has been previously described as “ghost epiphyses,” as these lines outline the “ghost” of the epiphysis at the time of the insult to the epiphysis, in case of our patient roughly the size and morphology expected in a female of 3 years old [8, 10]. Sparse case reports also refer to epiphyseal ghosts in the knee but used the description “intra-epiphyseal silhouettes” or “patellar growth arrest lines,” when occurring years after long periods of immobilization due to trauma or surgery in childhood [14, 15]. Other authors refer to the existence of epiphyseal growth arrest lines in their papers on imaging of pediatric bone growth [16, 17]. Because the acrophysis is not restricted to the epiphyses but is also seen in other SOC, including the patella, the term “ghost SOC” seems to be more appropriate.

We observed higher T1 and PD signal intensities with sparse trabeculation in our patients’ ghost SOC on MRI compared with normal-appearing signal intensities in the surrounding epiphyses as well as the absence of metaphyseal growth arrest lines. These observations were also described by Yao et al. in their case presentation of two adults, both suffering from long-term immobilization in their childhood due to trauma and treatment of developmental dysplasia of the hip respectively. They suggested that the relatively greater rate of bone remodeling in metaphyses might explain the disappearance of metaphyseal growth lines.

Thus, MRI shows evidence of failure of resumed normal bone modeling in the ghost SOC. We hypothesize that hitherto unraveled differences between physeal and acrophyseal endochondral bone growth processes as well as differences in modeling capacities of the physis and epiphysis might underlie these observations. A study on acrophyseal and physeal hypertrophic chondrocytes showing clear differences in the molecular composition of the same cells at both locations seems to support this hypothesis [18].

The persistent lack of trabeculation and the “empty” aspect of the ghost SOC might, therefore, well reflect the appearance of the SOCs at the time of the growth arrest or, even better, at the precise moment of growth recovery, immediately following the insult to the bone. The clear decrease in trabeculation in the ghost SOC of our patient as seen on radiographs as well as on MRI can be linked to her very low BMD, measured on DXA. Why normal bone modeling in the ghost epiphyses could not be resumed after mobilization, bisphosphonate therapy and recovery from the ALL, and normalization of BMD as a result remains unclear.

In summary, we describe a rare case of persistent acrophyseal growth arrest lines in a patient who suffered from ALL as a child, complicated by long-term immobilization and reviewed the sparse literature on reports of comparable cases. All previous reported cases suffered from long-term immobilization of the lower extremities in early childhood because of trauma or due to operative procedures. Multifactorial causes of bone morbidity might underlie these persistent “ghost” SOCs. This probably warrants further studies on biochemical processes underlying physeal and acrophyseal endochondral ossification.
